# Electrical stimulation in bone tissue engineering treatments

**DOI:** 10.1007/s00068-020-01324-1

**Published:** 2020-02-20

**Authors:** Liudmila Leppik, Karla Mychellyne Costa Oliveira, Mit Balvantray Bhavsar, John Howard Barker

**Affiliations:** Frankfurt Initiative for Regenerative Medicine, Experimental Orthopedics and Trauma Surgery, J.W. Goethe University, Frankfurt/Main, Germany

**Keywords:** Electrical stimulation, Bone regeneration, Bone tissue engineering, In vitro, In vivo

## Abstract

Electrical stimulation (EStim) has been shown to promote bone healing and regeneration both in animal experiments and clinical treatments. Therefore, incorporating EStim into promising new bone tissue engineering (BTE) therapies is a logical next step. The goal of current BTE research is to develop combinations of cells, scaffolds, and chemical and physical stimuli that optimize treatment outcomes. Recent studies demonstrating EStim’s positive osteogenic effects at the cellular and molecular level provide intriguing clues to the underlying mechanisms by which it promotes bone healing. In this review, we discuss results of recent in vitro and in vivo research focused on using EStim to promote bone healing and regeneration and consider possible strategies for its application to improve outcomes in BTE treatments. Technical aspects of exposing cells and tissues to EStim in in vitro and in vivo model systems are also discussed.

## Introduction

Bone is one of the few tissues in mammals, that when fractured “regenerates” on its own. However, in cases where large volumes of bone are missing, like in severe injury, surgical extirpation of large amounts of infected bone or tumors, and congenital skeletal abnormalities, these regenerative capabilities are overwhelmed and complex and costly treatments must be employed to close the defect. Among conventional treatment options, bone autografts are considered to be the gold standard. However, in spite of the success they enjoy, autografts are associated with drawbacks, like donor site morbidity, limited availability in overly large defects, and the risk of infection (reviewed in [1]), which continue to fuel the search for better, alternative treatments. Bone tissue engineering (BTE) has recently been introduced as an alternative to conventional treatments, for large non-healing bone defects, and holds great promise for promoting bone healing and regeneration without the associated drawbacks [2]. BTE approaches, in many ways, simulate bone autografts, in that they fill the defect with bone-forming stem/progenitor cells, scaffolds that restore missing bone volume, and growth factors that control cell–cell and cell–scaffold interactions [3]. Success of BTE approaches, in clinical settings, depends largely on the choice of cells, scaffold material, and signaling stimuli added to the cell–scaffold mix, and/or present in the microenvironment of the healing defect. While pre-clinical and clinical BTE treatments have demonstrated encouraging early outcomes [4, 5], the logistics associated with harvesting, isolating and amplifying the cells, and the time required to do so, are not optimal and continue to stimulate the search for strategies to manipulate/fine-tune the type, quantity, and composition of stem cells, scaffolds, and stimuli (reviewed in [3]).

For decades, electrical stimulation (EStim) has been studied and used successfully in clinical practice to stimulate bone healing (reviewed in [6]). While the detailed mechanisms by which EStim promotes healing are poorly understood, several recently published in vitro studies suggest that EStim’s pro-healing effect is due to its influence on the behavior and/or function of bone-forming stem cells, such as migration [7, 8], proliferation [9], differentiation [10, 11], mineralization [12], extracellular matrix deposition [13], and attachment to scaffold materials [14]. Importantly, all these cell behaviors/functions that are central to healing could potentially be used to optimize outcomes in BTE treatments. In this review, we provide an overview of different methods and results using EStim to treat cells, scaffolds, and tissues in in vitro and in vivo model systems, with an eye toward its potential use in BTE treatments. We discuss mechanisms by which EStim acts at cellular and molecular levels and discuss limitations and technical aspects of delivering EStim both in experimental and clinical settings. This knowledge could assist in the development of future clinical strategies for combining EStim and BTE treatments.

## Applying EStim in bone tissue engineering treatments

EStim could potentially be added in clinical BTE treatments either ex vivo, when the cell–scaffold construct is prepared, or in vivo, after the cell–scaffold construct is delivered into the bone defect.

## EStim’s effects on cell function

**Fig. 1:**
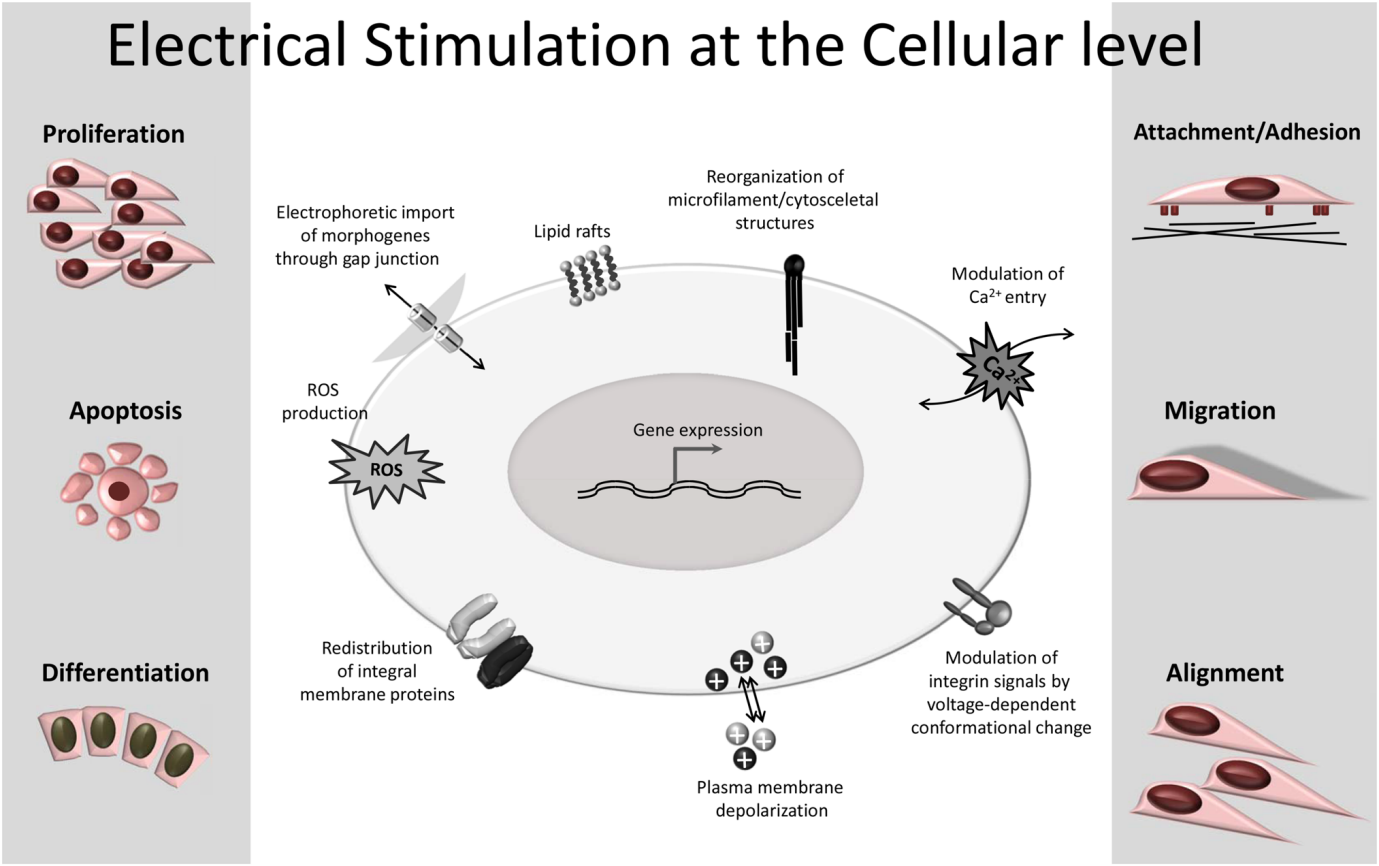
Cellular mechanisms and functions activated by EStim

Previous in vitro experiments that exposed cells and/or scaffolds to EStim, demonstrated its ability to influence cell functions associated with enhanced bone healing [7–14]. These experiments were conducted on a variety of different cell types; bone marrow-derived mesenchymal stem cells (BM-MSC), from human and animal origin [10, 11, 15–25]; adipose-derived mesenchymal stem cells (AT-MSC) [11, 20, 26–30], mouse osteoblast-like cells [31–33] and more recently, human dental pulp stem cells (DPSC) [34]. The above cell types are commonly studied for use in BTE applications. Based on these findings, one can speculate that treating cell–scaffold constructs with EStim ex vivo, prior to placing the mix in a bone defect, would greatly improve outcomes in BTE in treatments. How DC EStim affects these cells is summarized in the following paragraphs and Fig. 1.

### Cell proliferation and apoptosis

The number of stem/progenitor cells that can be obtained for use in BTE construct preparation is often limited by the amount of donor material that can be harvested, usually from bone marrow or adipose tissue. While possible, in vitro expansion of donor cells, to reach adequate numbers for therapeutic doses, is not an optimal solution, as it is time consuming and can negatively impact stem cell “quality” [35]. Although EStim has mainly been shown to increase the rate of cell proliferation, contrary findings exist, that show EStim can also decrease, or have no effect on cell proliferation (for general review refer to [36]). Our own experience showed that daily application of 50–150 mV/mm DC EStim has no effect on rat BM-MSC and AT-MSC proliferation, when cultured in 2D or 3D (with scaffolds) [11, 29, 37]. That said, others have shown that longer application of DC EStim enhanced rat BM-MSC [22] and human BM-MSC proliferation. In addition, using EStim, in the form of a degenerating sine wave, which deteriorates over time (degenerate wave—DW), Griffin et al. were able to show an even greater effect on cell proliferation [16]. When treating osteoblasts in static medium conditions, Kumar et al. describes DC EStim as having no or negative effects on cell proliferation [38]. Others have shown positive effects of DC EStim on proliferation in 3D in vitro studies with fetal human or neonatal rat osteoblasts [39–41].

The effect of EStim on cell apoptosis, which may accompany enhanced cell proliferation [42] is unclear, as some studies have reported enhanced effect, while others describe a decrease or no effect at all (for more details see [36]). In summary, EStim’s effect on cell proliferation and apoptosis appears to be heavily dependent on the type and origin of the cells, the stimulation regimen and culture conditions [16, 43].

### Cell differentiation

EStim has been shown to enhance MSC osteogenic differentiation in a number of studies. We and others have demonstrated that DC EStim stimulates osteogenic differentiation in rat BM-MSC and AT-MSC, cultured in osteogenic medium in both 2D and 3D (with scaffold) culture conditions [10, 11, 16, 29, 37, 44, 45]. Interestingly, recent studies showed that applying EStim, in the early stages of MSC osteogenic differentiation (first 7 days), is sufficient to induce a strong, sustained, and long-lasting pro-osteogenic effect [10, 45]. As it relates to BTE treatments, this approach would benefit the logistics of treatment, making it possible to pretreat cells + scaffold with EStim, ex vivo, prior to placing them in a bone defect. In theory, by triggering this sustained pro-osteogenic effect, EStim-pretreatment, would promote healing in the defect long after discontinuing its delivery. We are currently testing this hypothesis in ongoing in vitro and in vivo experiments in our laboratories.

### Cell alignment

Cell alignment plays a critical role in embryonic development, growth, and regeneration [46], as it provides specific hierarchy of cells’ physical and mechanical properties and biological functions at the tissue level (reviewed in details in [46]. As it relates to BTE treatments, cell alignment is critical in cell–cell and cell–scaffold interactions during osteogenic differentiation and mineralization. DC EStim has been shown to significantly affect cell alignment. Several in vitro 2D-culture studies report DC EStim causing MSC and osteoblasts to undergo retraction and elongation, ultimately resulting in the realignment of the long cellular axis perpendicular to the electric field [30, 44, 47, 48]. In in vitro 3D‐culture studies, Yang et al. describes EStim as “promoting synergy” between cells and scaffold material [48]. Finally, it was shown that not only cell alignment, but also cell division plane, could be controlled by externally applied EStim [49, 50], theoretically making it possible to control the direction of cellular expansion.

### Cell migration

Cell migration is a behavior that is essential in embryonic development, and in tissue growth and repair, and in BTE applications can play an important role in cell infiltration into scaffolds and integration with host tissues. When exposed to externally applied EStim, similar in magnitude to endogenous electrical fields, many types of cells migrate in specific direction, and the speed and direction of cell migration are voltage dependent (reviewed in details [51, 52]). The movement of cells along an electric field gradient, or electrotaxis, appears to be dependent on species and cell subtype differences. For example, cells of osteosarcoma cell line, SAOS, migrate in the opposite direction as rat calvaria osteoblasts [53, 54]. Interestingly, similar cells from different origin were shown to have different electro-kinetic properties. For example, AT-MSC display different traveling wave velocity and rotational speed compared to BM-MSC [55, 56].

### Cell attachment/adhesion

Cell attachment/adhesion is known to affect cell behavior and function. For example, osteogenic stem cell differentiation was shown to be positively influenced by strong adhesion to surfaces with rough microtopographies [57]. 3D scaffold material, upon which cells are seeded in tissue engineering applications, provides anchorage for cells and are said to create a microenvironment which promote cell differentiation, metabolic activity, and cell–cell signaling [58]. The positive effect of EStim on cell–scaffold attachment has been demonstrated and described by several groups in several different in vitro experimental protocols [39, 59, 60]. In the rapidly growing field of smart biomaterials, scaffolds, made of electrically active biomaterials, are specifically designed to deliver EStim to cells, to promote tissue formation (reviewed in details in [61]).

In summary, at this point, the incorporation of EStim into BTE treatments, is mostly at the in vitro stage of development, and can potentially make it possible to fine-tune cell alignment, cell division, differentiation, migration, and attachment to scaffolds. These in vitro studies are laying the groundwork for subsequent in vivo studies that can be used to optimize outcomes in future BTE clinical studies.

## EStim-induced cell response—mechanisms

Exposing cells to exogenous EStim generates a response called electrocoupling, caused by high resistance of the plasma membrane, which prevents the penetration of electric stimuli, independent of the cytoplasm conductive capacity [62]. One of the possible electrocoupling mechanisms involves asymmetric redistribution/diffusion of electrically charged cell membrane receptors in response to electric fields, which further activates numerous downstream signaling cascades. Another possible mode of action is related to the cell membrane depolarization due to direct activation of voltage-gated Ca^2+^ channels, which leads to increase in intracellular calcium ion concentration, a cellular response consistently reported after electric stimuli. These and other mechanisms are discussed with more details below.

*Signal transduction pathways* Electrical signals are sensed and converted into biochemical cues by multiple pathways within cells, resulting in various biological responses. The activation of the MAPK (mitogen-activated protein kinase) cascades represents a major signal transduction pathway, which regulates specific mRNAs transcription as consequence of external stimuli [63]. This leads to the activation of extracellular signal-regulated kinase ERK1/2 and 5, JNK and p38MAPK, that consecutively intervenes in important cell activities, such as proliferation, differentiation, apoptosis and others, depending on the type of cell and stimuli [64, 65]. Fast and sustained phosphorylation of extracellular signal-regulated kinase (ERK), p38 mitogen-activated kinase (MAPK), Src and Akt, was demonstrated by Zhao et al. in cells migrating under the influence of electrical fields [66, 67]. EStim was shown to induce direction and movement of adult stromal cells through the activation of PI3K and ROCK signaling pathways [59].

*Ca*^*2*+^ *transients* Increase in intracellular Ca^2+^ is one of the prompt effects of EStim on cellular response. Calcium ions are important cellular mediators, which play a role in many important vital activities such as proliferation, differentiation, and apoptosis [68]. Intracellular Ca^2+^ could be increased via two essential events; by the passage of extracellular Ca^2+^ into intracellular space through plasma membrane ion channels, or by activation of specialized receptor/channels on the surface of the endoplasmic reticulum (ER), which release Ca^2+^ from internal stores in the ER [69]. Calcium oscillations can increase the efficiency and specificity of gene expression, which guides the direction of cell differentiation [70, 71]. EStim was shown to facilitate differentiation of hMSC by changing Ca^2+^ oscillation patterns to patterns similar to those seen in osteoblasts [72]. Of note, EStim exposure can directly stimulate L-type voltage-gated Ca^2+^ channels (VGCCs) in the plasma membrane [73] that can elicit many regulatory responses through the enzymatic action of the Ca^2+^/calmodulin-dependent nitric oxide synthases [74].

*Mechanotransduction—cytoskeletal reorganization and actin distribution* Mechanotransduction is the conversion of external mechanical stimuli into intracellular electrical or chemical signals [75]. The inverse effect of mechanotransduction is the transformation of electrical stimuli into mechanical activity that causes tension in the cytoskeleton due to reorganization of the cytoskeletal filaments and actin redistribution. Changes in the actin structure could occur as consequence of interactions between plasma membrane and electrical stimuli [44]. Hereof, EStim has been shown to cause either direct effects on the cytoskeleton, or intervene on cellular processes regulated by the cytoskeleton [76].

*Surface receptor redistribution* Most likely, DC EStim is restrained by cellular plasma membrane, which holds high electrical resistance, and events take place at the cell surface rather than penetrating inside the cell. As a result, most of the biochemical signal transduction cascades in response to EStim, arise due to the redistribution of charged cell surface receptors (CSRs) at the external space of cell membrane [77]. It is reported that the exposure to 100–3000 mV/mm of external EStim results in redistribution membrane proteins and lipids on external site of the cell due to induction of relative electrophoretic movement these components on the cell exterior [78]. Specifically, epidermal growth factor receptor (EGFR) was shown to be up-regulated by the application of low levels of EStim, which also induces EGFR redistribution and accumulation at the cathode side of the cell [79]. In addition to promoting an asymmetric distribution of EGFR, colocalization of membrane lipids and second-messenger signaling molecule ERK ½ could also occur due to influence of small amounts of EStim, resulting on the triggering of MAPK signaling cascade [49, 79].

*ATP synthesis* Direct current EStim, ranging from 10 to 1000 µA, is known to stimulate membrane-bound ATP synthesis [80]. This is thought to be due to EStim guiding migrating protons to reach the mitochondrial membrane-bound H1-ATPases, to generate ATP. This is supported by the observation of high levels of released ATP measured in electrically stimulated cells [81]. The relationship between ATP synthesis and actin cytoskeleton is one of the intriguing mechanisms to explain how cells sense electrical fields. It has been well documented that intracellular ATP is consumed for the conversion of monomeric G-actin to polymeric F-actin [82] and that EStim-induced ATP depletion is implicated in the reorganization of actin cytoskeleton in electrically stimulated hMSC [76].

*Heat shock proteins* It has been generally hypothesized that cells’ response to EStim (especially at levels higher that those occurring naturally) could follow physiological stress response and function through activation of stress heat shock proteins [83]. The involvement of heat shock proteins (hsp 27 and hsp 70) in the upregulation of some of the transcription factors was previously reported in hMSC osteogenic differentiation [84].

*Reactive oxygen species* Participating in crucial signaling pathways, reactive oxygen species (ROS) is considered another important mechanism involved in stem cell response to EStim [85]. Controlled induction of ROS at physiologic levels can benefit interactions of other signaling molecules influencing differentiation. Numerous studies have demonstrated that MAPK pathways and the subsequent signaling cascades of ERK1,2, JHK, and p38 are activated by moderate levels of ROS [86]. Proliferation and differentiation of MSC were shown to be mediated by a mild rise in hypoxia-induced ROS [87–89].

*Lipid rafts* Recent research has shown that in addition to proteins, lipids in the cellular membrane also participate in the response to externally applied EStim. It was shown that due to externally applied EStim, plasma membrane glycolipids could redistribute and congregate into nanodomain structures, known as lipid rafts [90]. Acting as the initial sensor of electric fields, these nanodomain structures polarize, coalesce, and segregate membrane proteins, which in consequence trigger intracellular signaling events to guide cell migration [91].

All these cellular mechanisms are involved in a complex and finely orchestrated network of signaling and responses. Biological processes are programmed as a chain reaction starting from a cellular activity, which normally leads to implications in the tissues they compose. Therefore, it follows that external interferences in the cell response, promoted by the application of EStim, influence not only cells but also tissues as well.

## Effects of EStim on bone healing

Bone healing is a complex and well-orchestrated process, both in time and space, requiring coordinated function of different cell types and systems [92]. EStim’s ability to promote bone healing has been demonstrated both in animal experiments and clinical settings (reviewed in [6]). When EStim is applied to a bone defect, alone or in combination with BTE constructs, its effect on all the resident cells needs to be considered. In the following lines we discuss how EStim, when added to BTE treatment might influence key bone healing parameters, like osteogenesis, vascularization, and inflammation.

### Osteogenesis

The positive effect of EStim on osteogenesis is well documented in numerous in vitro and in vivo studies, and in clinical applications (reviewed in [6]). In our own in vivo studies, we recently showed that DC EStim, when applied in combination with BTE treatment [29] resulted in significant new bone formation, by stimulating MSC proliferation and differentiation. The underlying mechanism suggested for this effect is that EStim enhances bone healing by stimulating the calcium–calmodulin pathway secondary to the upregulation of bone morphogenetic proteins, transforming growth factor-β and other cytokines (reviewed in [93]; [29, 94]).

### Chondrogenesis

Endochondral ossification plays a pivotal role in bone healing. During this process, progenitor cells differentiate into chondrocytes which later undergo maturation and mineralization, finally resulting in new bone tissue (reviewed in [95]). Whereas the positive effects of EStim on endochondral ossification have been shown in previous studies [29, 96, 97], there few studies focused on analyzing the effects EStim has on endochondral ossification and MSC chondrogenic differentiation (reviewed in [98]). In our own in vitro studies, we were not able to detect a positive effect of direct current EStim on rat MSC chondrogenic differentiation (unpublished data). However, others have reported in vitro studies using pulsed EStim that showed the opposite (reviewed in [99]. One study showed that EStim alone stimulated MSC chondrogenic differentiation [23], and others showed that only applying an electrical field together with a chemical inducer (transforming growth factor-β3) induced MSC chondrogenic differentiation [100, 101]. A new interesting approach that applies nanosecond pulse EStim treatment to potentiate MSC chondrogenic activity was recently reported and showed encouraging preliminary results both in vitro and in vivo [99, 102]. Although these recent examples suggest that EStim has a positive effect on chondrogenesis, additional studies are needed to confirm this effect and to sort out the underlying mechanisms.

### Vascularization

New vessel formation plays a pivotal role in all forms of healing and regeneration and BTE is no exception [103]. In the case of defects that require large volumes of cells and scaffold material, once the construct is placed in the defect, its innermost part does not receive adequate vascularization causing ischemia and cell death in the graft. A number of studies have been performed that test different methods of stimulating new vessel formation into the tissue engineered constructs (reviewed in [104]). Several studies, in dermal wounds, have demonstrated EStim’s ability to stimulate new vessel ingrowth from pre-existing blood vessels in adjacent tissues into ischemic wounds [105–109]. DC EStim was shown to promote important angiogenic responses of vascular endothelial cells and selectively regulate production of growth factors and cytokines important in angiogenesis through a feedback loop mediated by VEGF receptors [110, 111]. In our own studies, in a rat femur large defect model, adding EStim to BTE-treated bone defects caused a significant increase in new vessel formation into the defect [29].

### Inflammation

It is well known that bidirectional cross talk between immune cells and bone cells is crucial for bone remodeling and repair [112]. The close interaction between the immune system and bone healing is well documented. In the emerging research field of osteoimmunology, the early inflammatory phase of healing is a promising target for immunomodulatory approaches to enhance bone healing [113]. Even though the role of immune cells and cytokines in bone healing has been recognized for 20 years now, and EStim has been used to treat bone fractures even longer, little is known about the effects of EStim on immune cells during bone healing.

The immune system plays a crucial role as the host’s first responder following injury, in which case macrophages are rapidly recruited to the site of injury initiating the inflammatory response [114]. Whereas early studies showed that DC EStim does not alter macrophage phenotype [115], more recent studies describe EStim causing macrophages and monocytes to migrate away from the stimuli. Moreover, EStim was shown to significantly enhance macrophage phagocytic uptake and to selectively modulate cytokine production [116]. EStim’s effect of upregulating osteogenic gene (Spp2 and Bmp2) transcription in macrophages could help explain its role in stimulating osteogenesis [89]. In vivo*,* low-voltage EStim was shown to modify macrophage response by changing the M1 to M2 macrophage ratio [97]. Overall, these findings suggest that EStim can exert a significant effect on these macrophage sub-populations. The goal of ongoing studies is to use EStim to fine-tune the response of macrophages, and other immune cells from pro-inflammation to pro-regenerative in BTE treatments.

## EStim in bone tissue engineering treatments—current status and limitations

There are a growing number of studies in the literature that focus on combining EStim and BTE treatments [117]. In addition to studying how best to use EStim to manipulate cell behavior, it is also important to consider technical aspects of delivering EStim to cell + scaffold constructs and/or tissues in clinical treatments. EStim devices for use in treating ex vivo cells prior to transplantation will have to be developed, while commercially available DC bone growth stimulators (OsteoGen® from Biomet EBI and Zimmer direct current bone growth stimulator, reviewed in [118, 119]) could be adapted for treating transplanted BTE constructs in clinical settings.

## Applying EStim in vitro

**Fig. 2:**
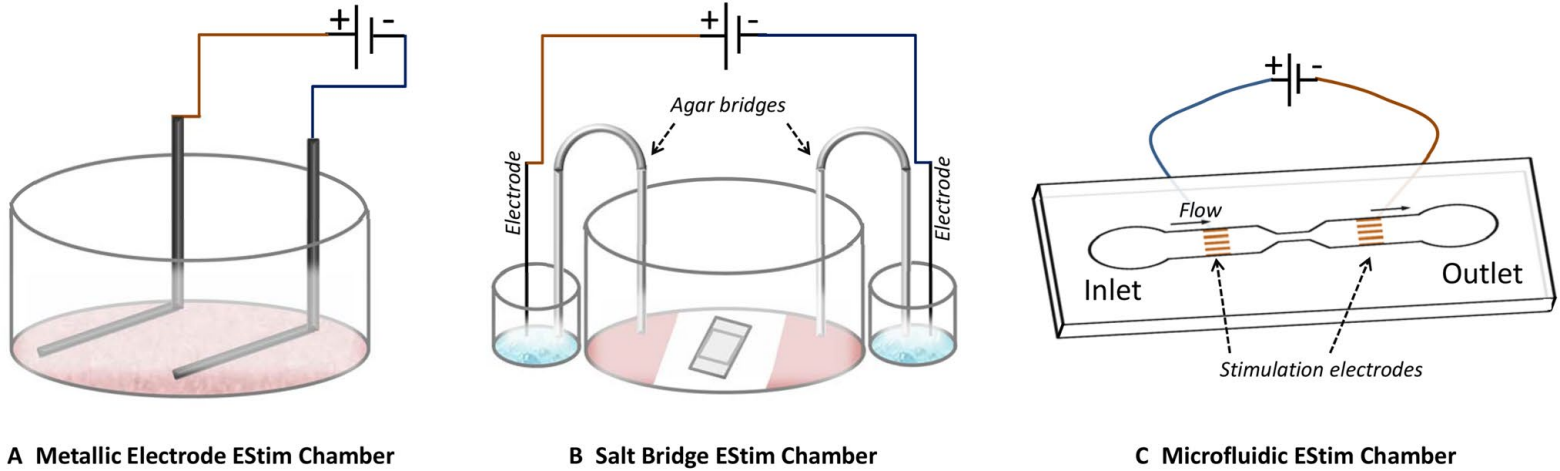
EStim setups commonly used to stimulate cells in vitro*.***a** Metallic electrode EStim chamber delivers EStim to cells via metallic electrodes submerged directly in culture medium in standard cell culture plates. **b** Salt bridge EStim chamber delivers EStim to cells through salt bridges submerged in culture medium. **c** Microfluidic EStim chamber, uses micropumps to move cells in and out of constricted channels where they are trapped and exposed to EStim

In most in vitro applications, cells grown in 2-D or 3-D culture can be treated with specific regimens of EStim in purpose-built chambers. As these chambers are not commercially available, different laboratories have developed their own to satisfy their specific needs. Below, we describe a few of the most commonly used setups (Fig. 2).

### Metallic electrode EStim chamber

Perhaps the simplest in design and to use, are chambers in which EStim is delivered directly to cells in culture by means of metallic electrodes. Different types of metals are used for the electrodes, including stainless steel [120], copper [121], platinum [122, 123], silver/silver chloride [124], iridium oxide, and titanium nitride [125]. Although platinum is inferior in stiffness to other metals and is the most expensive, it is nevertheless preferred over other metals since it is less prone to corrosion [126]. Generally, one end of the electrodes is bent to fit into a cell culture well where they are submerged into culture medium containing the cells, and the other end of the electrodes is connected to a power supply.

Standard 6-, 12- or 24-well cell culture plates are used, and the electrodes are attached to removable lid(s) or inserted directly into the well(s) [37]. This type of setup has the advantage that multiple samples (wells) can be stimulated simultaneous, thus increasing reproducibility. The voltage range deliverable with this setup is generally from tens to hundreds of mV/mm. For example, in one of our studies using this setup, we exposed rat MSC to 100 mV/mm of DC EStim for 1 h/day, for 7–21 days, and demonstrated that this EStim regimen improved mineralization and expression of osteogenic marker genes [10, 11, 37]. In another study, Wang et al. showed that 200 mV/mm of DC EStim for 4 h enhanced migration, proliferation, and differentiation of rat BM-MSC [22].

The main advantage of this type of EStim chamber is that it is a simple design that does not require special equipment/knowledge to build and use. A detailed video demonstration of how to build and use this type of EStim chamber is available at [122]. A drawback associated with this type of setup is the possible generation of cytotoxic faradic products on the electrode surface, which limits the duration and intensity of EStim that can be applied to the cells [37, 122]. The addition of a peristaltic pump to the culture plate(s) that regularly exchanges culture medium would avoid these limitations [38].

### Salt bridge EStim chamber

Another commonly used EStim chamber delivers EStim to cells through salt bridges submerged in the culture media. The salt bridges separate the culture medium, and cells from the metallic electrodes thus preventing them from being exposed to cytotoxic electrochemical byproducts and pH changes [127]. Salt bridges contain a saturated solution of inert salt, usually NaClO4, KCl, or KNO3. These act as electrochemical cells that work like batteries, transferring electric current to ionic current through the salt bridges via redox reactions [128]. The voltage required using salt bridge EStim chambers is relatively large, around 70 V, to overcome the resistance of the bridges [124].

While the salt bridge setup/method has been widely used to study the effects of EStim on cultured cells, it has a number of limitations: (1) small working area limiting the number of cells that can be studied in a single setting; (2) limited EStim exposure time due to the concentration and heat differences between the bridge contents and the media; (3) technically complicated to set up and run experiments, making sterility and reproducibility a challenge; (4) setup differs significantly from metallic electrode stimulators used in vivo and in clinical settings; therefore, the correlation and consequently interpretation between in vitro and in vivo study results are problematic.

### Microfluidic chip EStim chambers

In the EStim chamber designs described above, the cells are often exposed to toxic electrolysis products and the electrical field generated is not homogenous. These limitations overcame in microfluidic chip EStim chambers [129–132]. Microfluidic EStim chambers consist of (1) an inlet for loading cells, (2) a main fluidic channel, (3) a constriction microchannel/microchip, (4) a pair of stimulation electrodes for applying electrical stimulation and reference electrodes for measuring extracellular field potential simultaneously, and (5) an outlet reservoir for collection of cells after EStim [133]. To use this chamber, cells are first loaded through the inlet, then, by controlling the driving pressure of the flow and using a constriction channel, cells are trapped on the surface of measurement electrodes, where they are exposed to EStim. After stimulation, cells are driven to an outlet where continuous measurements are performed. The small cross section of the chamber limits the amount of electrical current applied and reduces the cytotoxic products that can harm the cells. Some limitations of microfluidic chip EStim chambers are that their small size requires that they be specially manufactured, the setup procedure prior to running an experiment is complicated and their small size results in low cell yield and poor cell product recovery [132].

While the EStim chambers described above are relatively well suited to the needs of researchers for conducting in vitro experiments to study the effects of EStim on different cell/scaffold combinations, their use for preparing large biomimetic BTE constructs for transplantation in vivo or ex vivo in a clinical setting is not adequate. For this, special EStim chambers will have to be developed/adapted to accommodate clinical requirements such as scaling up (size adaptation), on line monitoring, standardization, sterilization, and cost considerations. To grow and cultivate engineered bone constructs long term, biomimetic perfusion bioreactors are under development that take into consideration flow of culture medium and fluid-shear stress, position specific oxygen gradients, mechanical, and physical stimulations [134–136]. These adaptations could allow direct comparisons between in vitro studies, move the above mentioned exciting new laboratory findings closer to in vivo applications and closer to the ultimate goal of clinical application in BTE treatments.

## Clinical EStim bone treatment devices

**Fig. 3:**
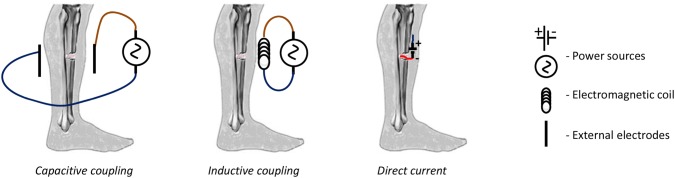
Clinical EStim devices. External stimulators deliver capacitive coupling and inductive coupling (pulsed electromagnetic field) EStim, and internal stimulators deliver direct current (DC) EStim via surgically implanted device

There are a number of commercially available clinical EStim devices that could be used/adapted to treat BTE constructs with EStim, before they are loaded into bone defects. Devices used for EStim bone treatment in clinical settings, can be categorized into external and internal stimulators, that deliver EStim via an external field or percutaneously, and via internal surgically implanted electrodes. The external stimulators deliver capacitive coupling (CC) and inductive coupling (Pulsed ElectroMagnetic Field—PEMF) EStim, and the internal stimulators deliver direct current (DC) EStim [137, 138] (Fig. 3).

*Capacitive coupling* stimulators are small, lightweight devices, which use an external power source. Despite the obvious advantages of not having to be surgically implanted, and ease of use, disadvantages include, patients must change batteries daily, skin irritation, and patient non-compliance are common problems [138]. There are only a few clinical studies available that support the effectiveness of CC devices (reviewed in [6]).

*Inductive coupling or pulsed electromagnetic field (PEMF):* The electrodes of these devices can be placed under casting material or used through a cast. These devices create low-level electromagnetic signals, which after reaching the fracture site, are converted into electric currents and are said to mimic the body’s normal physiologic processes. The primary advantage of PEMF bone stimulators is their noninvasive application; however, drawbacks include, the heavy weight of these devices, difficulty assessing treatment dosage, and patient non-compliance [139].

*DC electrical stimulators* have the benefits of providing constant and uniform current delivery, the EStim is focused at the bone defect, and elimination of patient non-compliance. Surgical implantation consists of placing a cathode at the fracture site and an anode in the nearby subcutaneous tissue, that deliver electric current flow between them. The electrodes are connected to a stimulator device, which is implanted subdermally [140, 141]. The power source of these devices typically last from 6 to 8 months, at which time the implanted device and electrodes must be removed in a second procedure. Over the last 3 decades, numerous studies support the clinical efficacy of these DC EStim stimulators [138, 142, 143]. Recent clinical studies using implantable DC EStim stimulators, alone [144], or in combination with bone grafts [145] have reported increased bone healing rates.

Other physical stimulation techniques, like magnetic and vibration stimulations, have been tried and found to promote bone healing [146], in general their administration in patients in clinical settings have come up against serious limitations. The wearable devices used for their administration are cumbersome, thus when used for prolonged periods tends to interfere with patients’ daily activities leading to decreased compliance [6]. In addition, these units have been reported to give inconsistent results, and one of the reasons for this has been attributed to the fact that the stimulation energy they generate is not focused at the fracture site. In contrast, when DC EStim is administered with surgically implanted device compliance is not an issue and the electrical energy is focused at the fracture site, which has led to the reporting of more consistent treatment outcomes.

Despite EStim’s demonstrated effectiveness improving bone healing, both in pre-clinical animal studies and in clinical settings, few studies have investigated the effectiveness of combining EStim with BTE treatments in vivo [27, 29, 94, 147, 148]. In one of these studies, our group treated large defects in rat femurs with AT-MSC + Scaffold + EStim. We found that the rate and quality of bone healing at 8 weeks, in defects treated with AT-MSC + Scaffold + EStim, was significantly better than controls [29]. Our own experience from this study, together with reports in the literature [141, 149], suggests that the problems of combining EStim with BTE in clinical settings would be similar to those experienced in current clinical EStim bone treatments. Namely, complications associated with the surgical procedures used to implant and explant the EStim device, electrode breakage or dislodgement, and infection.

## Current developments

Summarizing, in vitro experiments that expose cells and/or scaffolds to EStim, generally show positive results, although, a lack of standardization of cell types, models and protocols make it difficult to draw definitive conclusions. Additional studies are needed to develop strategies for transferring these encouraging in vitro findings into meaningful in vivo BTE applications. In addition, the logistics of combining EStim and BTE treatments in practical, cost effective ways in clinical settings must be considered.

Some exciting new developments that could be incorporated into these strategies include the use of electroactive smart polymeric biomaterials that could potentially combine scaffold and EStim into one. Recent advancements in polymer science, using “smart” biomaterials, that enable built-in stimulus/response behavior capabilities, have tremendous potential [150]. Electroactive smart polymeric biomaterials could be used to build scaffolds that offer precise control over the amount, duration, and localization of the electrical stimulus, thus obviating the need for bone stimulators. These materials have already been tested in vitro*,* where they have demonstrated the ability to improve cell proliferation and differentiation [151–153]. If in addition to promoting these pro-osteogenic activities, these smart biomaterials can also be designed to biodegrade after a given useful time period, this would be yet another benefit [154].

## Conclusions

EStim has the demonstrated ability to improve osteogenic potential in various types of MSC and osteoblast-like cells in vitro*,* and to stimulate new tissue formation like, bone, cartilage, and vessels in vivo. It is important to recognize that these encouraging early findings are strongly dependent of many factors like type and origin of cells, EStim regimen, and area of the defect to be treated. The variability of these results reported in the literature make it difficult to compare and develop a single optimal EStim + BTE protocol. Accordingly, it is important that future in vitro experiments be planned and conducted with an eye toward applying the findings in in vivo models and regimens that can be transferred into to clinical protocols. Combining EStim and BTE treatments has the potential to create synergies that could result in outcomes that far exceed those achieved by either treatment on its own.
